# Comparative Mitogenomics and Phylogenetics of the Nose Flies (Diptera: Calliphoridae, Rhiniinae)

**DOI:** 10.3390/ani16091289

**Published:** 2026-04-22

**Authors:** Tingying Li, Krzysztof Szpila, Arianna Thomas-Cabianca, Thomas Pape, Xingkun Yang, Liping Yan, Dong Zhang

**Affiliations:** 1 Key Laboratory of Non-Invasive Research Technology for Endangered Species, School of Ecology and Nature Conservation, Beijing Forestry University, Beijing 100083, China; tingyingli@bjfu.edu.cn (T.L.); xingkun_yang@bjfu.edu.cn (X.Y.); 2 School of Biological Sciences and Technology, Beijing Forestry University, Beijing 100083, China; 3 Faculty of Biological and Veterinary Sciences, Department of Ecology and Biogeography, Nicolaus Copernicus University in Toruń, 87-100 Toruń, Poland; szpila@umk.pl; 4 Senckenberg Natural History Collections Dresden, 01109 Dresden, Germany; arianna.thomas-cabianca@senckenberg.de; 5 Natural History Museum of Denmark, University of Copenhagen, DK-2100 Copenhagen, Denmark; tpape@snm.ku.dk

**Keywords:** blowfly, evolution, insect, mitogenome, phylogeny, Rhiniinae, systematics

## Abstract

The Rhiniinae (Diptera: Calliphoridae), a recently reclassified subfamily of blowflies, comprise approximately 400 species across 30 to 39 genera, which occupy diverse ecological associations, including flower visitation and specialized associations with social insects, yet their phylogenetic relationships remain poorly established due to lack of molecular data. We sequenced and characterized the complete mitochondrial genome of six representative Rhiniinae species, with which the phylogenetic analyses were conducted. The monophyly of Rhiniinae was robustly supported and the internal relationships were clarified. Rhiniini and Cosminini were both recovered as well-supported monophyletic tribes using comprehensive mitogenomic evidence for the first time. In contrast to the purifying selection prevailing in most protein-coding genes, the COII gene showed consistent signatures of positive selection, potentially linked to the functional optimization of cytochrome c oxidase. Overall, this study provides foundational mitogenomic data and a robust phylogenetic framework, offering valuable resources for future research on mitochondrial evolution and systematics within this ecologically intriguing lineage.

## Simple Summary

Rhiniinae (Diptera: Calliphoridae) is a diverse group of blowflies with highly specialized breeding strategies. The phylogenetic relationships within this subfamily have remained unresolved, in part because molecular sampling has been limited. In this study, the complete mitogenomes were sequenced and annotated for six species of Rhiniinae, and their phylogeny was reconstructed. The monophyly of Rhiniinae was strongly supported; the two tribes Rhiniini and Cosminini were each recovered as monophyletic; and the genus-level relationships were inferred with molecular data for the first time. The COII gene was found to have evolved under strong positive selection according to comparative mitogenomic analysis.

## 1. Introduction

Rhiniinae, also known as nose flies, were recently reclassified from family rank as a subfamily of the Calliphoridae based on phylogenomic evidence [1]. With roughly 400 described species across 30 to 39 genera [2,3,4,5], they reach the highest diversity in the Afrotropical region, with further representatives in the Palaearctic, Oriental, and Australasian realms [5]. Although the biology of most species remains poorly understood [6,7,8,9,10,11], the known ecological traits are diverse; for example, adults in several genera are known as flower visitors, and larvae of some species exhibit associations with social insects [5,6]. Sophisticated morphological and chemical mimicry have been developed in larvae of a *Rhyncomya* species, as well as genera previously assigned to Prosthetosomatinae, to integrate into termite colonies [12], indicating the potential for highly specialized symbioses within the group. These diverse and intriguing life habits highlight the importance of establishing a robust phylogenetic framework to understand the evolution of such complex ecological interactions.

Typical insect mitogenomes are double-stranded circular molecules with compact size (~15–18 kb), maternal inheritance, and conservation in gene arrangement and genetic composition. Given the relatively high evolutionary rates and rare recombination, mitochondrial genomes have been widely used for phylogenetic inference of various insect groups [13,14,15,16,17,18,19,20,21,22,23]. These properties make mitogenomes especially valuable for clarifying both deep relationships and recent ecological adaptations in various insect groups [14,24,25]. However, although a good number of mitogenomes of Calliphoridae have been sequenced with the development of high-throughput sequencing, the data are biased toward several subfamilies, for example, Calliphorinae, Chrysomyinae, and Luciliinae [26,27].

Comparative analyses of calliphorid mitogenomes have revealed a generally conserved gene arrangement and intense purifying selection governing the evolution of most mitochondrial protein-coding genes [28]. Nevertheless, several studies have detected signatures of positive selection in oxidative phosphorylation-related genes, implying lineage-specific adaptive evolution underlying mitochondrial energy metabolism [29,30]. Despite these findings, the evolutionary drivers and functional consequences of this positive selection remain largely elusive.

To improve our understanding of the diversity in the mitogenomes and the evolution of the Rhiniinae, we sequenced and characterized the mitogenomes of six species. Comparative analysis was conducted among these newly sequenced mitogenomes and other mitogenomes of the Calliphoridae from GenBank [31], and subsequently, the phylogeny of blow flies was reconstructed with these mitogenomic data.

## 2. Materials and Methods

### 2.1. Mitogenome Acquisition and Taxon Sampling

Specimens of six Rhiniinae species representing the two recognized tribes were used for DNA extraction: *Isomyia tristis* (Bigot), *Metallea albifacies* Dear, *Rhyncomya callopis* (Loew), and *Rhyncomya pruinosa* Villeneuve from the tribe Cosminini, and *Rhinia nigricornis* (Macquart) and *Stomorhina rugosa* (Bigot) from the tribe Rhiniini. Collection information for all specimens is provided in Supplementary Table S1. Genomic DNA was extracted from the entire body of adult specimens preserved in 99.5% ethanol using the QIAamp^®^ DNA Micro Kit (Qiagen, Beijing, China, 56304), following the manufacturer’s instructions. The extracted DNA was then subjected to high-throughput sequencing on the Illumina NovaSeq 6000 platform (Gene+, Beijing, China), generating 150 bp paired-end reads for each sample. The mitogenomes were assembled and annotated using MitoZ 3.6 [32] with the SPAdes assembler, and the k-mer size was set to 71. The assembling was carried out under the Arthropoda clade setting, adopting the invertebrate mitochondrial genetic code. A circular map of each mitogenome was subsequently generated using the online server CGView (https://proksee.ca/, accessed on 1 June 2025).

Mitogenomes of calliphorids sensu lato were harvested from GenBank for comparative and phylogenetic analyses (Table 1). The unannotated mitogenomes from GenBank were annotated by aligning the mitogenomes with the protein-coding genes (PCGs) and ribosomal RNA (rRNA) genes of calliphorid references using Geneious Prime 2025.0.2 [33] following [28]. To improve the taxon sampling of phylogenetic analysis, species of Rhiniinae with mitochondrial genes available from GenBank were also included (Table 1; Supplementary Table S2).

### 2.2. Comparative Mitogenomic Analysis

Mitogenomes of Calliphoridae accessible in GenBank as of April 2025 were retrieved and analyzed, together with the six newly sequenced mitogenomes in this study (Table 1). The base composition and codon usage of the PCGs were analyzed utilizing MEGA X [34]. To evaluate mitochondrial strand asymmetry, the AT skew and GC skew of the 13 PCGs were computed using the following equations: AT-skew = (A − T)/(A + T) and GC-skew = (G − C)/(G + C).

The relative synonymous codon usage rates (RSCU) were calculated in the context of maximum likelihood phylogenetic analysis, using the online tool provided by GenePioneer Bioinformatics (http://cloud.genepioneer.com:9929, accessed on 27 July 2024).

Further comparative analyses, such as Pi values, nonsynonymous substitutions (Ka) and synonymous substitutions (Ks) were calculated to estimate the nucleotide diversity and evolutionary rate between subfamilies of Calliphoridae. The analyses were conducted with 54 species from four subfamilies, Calliphorinae, Chrysomyinae, Luciliinae, and Rhiniinae, for which there was more than one available mitogenome. MAFFT 7 [35] was used to align each mitochondrial gene with default parameters. The resulting alignment was then used to calculate Pi values and *Ka/Ks* ratios for each shared gene with DnaSP 6 [36].

### 2.3. Phylogenetic Analysis

Phylogenetic reconstruction was conducted using mitogenomes of Calliphoridae, with other oestroid and muscoid species as outgroups, together with mitochondrial genes of Rhiniinae harvested from GenBank (Table 1), to improve taxon sampling.

The PCGs and rRNA genes were aligned separately using MAFFT 7, and then concatenated using Sequence Matrix 1.8 [37]. The concatenated matrix was then used to infer phylogeny via maximum likelihood (ML) on the IQ-TREE web server [38]. The best tree was searched with the optimal model for each partition identified by the built-in model selector [39], and branch support was estimated through 1000 ultrafast bootstrap replicates. Bayesian inference (BI) was performed using MrBayes v3.2.7 [40] for 8,000,000 generations, sampling every 1000 generations. A consensus tree was constructed after discarding the first 25% of trees as burn-in. The phylogenetic tree was then visualized using iTOL 5 [41].

## 3. Results

### 3.1. General Features of Mitogenomes

**Figure 1:**
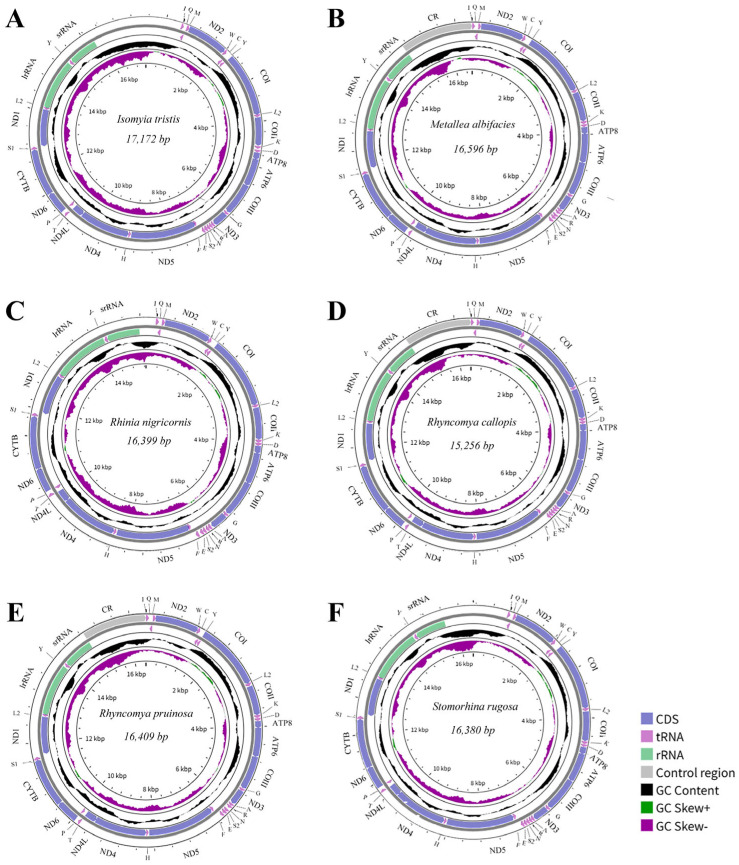
Mitogenome maps of *Isomyia tristis* (**A**), *Metallea albifacies* (**B**), *Rhinia nigricornis* (**C**), *Rhyncomya callopis* (**D**), *Rhyncomya pruinosa* (**E**), and *Stomorhina rugosa* (**F**). The strands are marked with arrows that indicate the direction of gene transcription. The IUPAC-IUB single-letter amino acid codes assign one-letter symbols to tRNA genes. Gene names are represented by their abbreviations: COI–COIII, cytochrome oxidase subunits 1–3; CYTB, cytochrome b; ND1–6 and ND4L, NADH dehydrogenase subunits 1–6 and 4L; ATP6 and ATP8, ATP synthetase subunits 6 and 8; lrRNA and srRNA, large and small rRNA subunits; CR, control region.

The complete or nearly complete mitogenomes of six Rhiniinae species were successfully sequenced for the first time: *I. tristis* (17,172 bp), *M. albifacies* (16,596 bp), *Rhi. nigricornis* (16,399 bp), *Rhy. callopis* (15,256 bp), *Rhy. pruinosa* (16,409 bp), and *S. rugosa* (16,380 bp) (Figure 1). *Rhi. nigricornis*, *Rhy. pruinosa* and *S. rugosa* were recovered as complete circular molecules, whereas the remaining three were nearly complete, with missing regions restricted to portions of the control region. The length variation is primarily attributed to the different sizes of the control region. The gene composition is consistent with that of the typical insect mitogenome, comprising 13 PCGs, two rRNA genes, and 22 transfer RNA (tRNA) genes. Four PCGs (ND1, ND4, ND4L, and ND5), two rRNAs (srRNA and lrRNA), and seven tRNAs (*trnV*, *trnP*, *trnH*, *trnF*, *trnY*, *trnC*, and *trnQ*) are encoded by the minority strand (N-strand). The remaining genes, including nine PCGs and 15 tRNAs, are encoded on the majority strand (J-strand).

**Figure 2:**
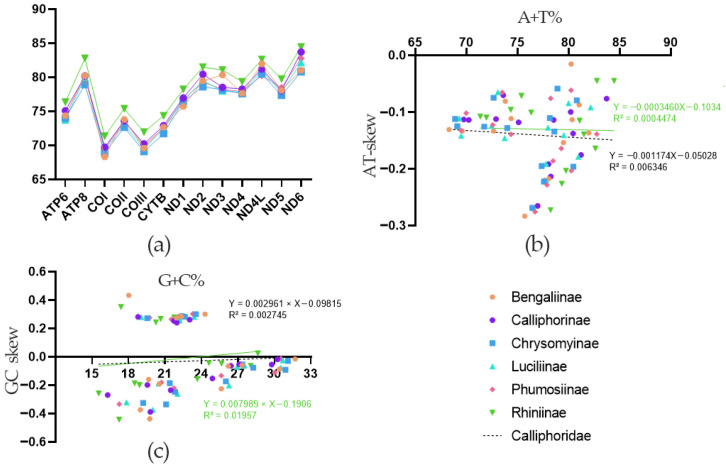
Nucleotide composition of mitogenomes from six subfamilies of Calliphoridae. (**a**) A + T percentage (AT%) of the 13 protein-coding genes; (**b**) correlation between AT% vs AT skew; (**c**) correlation between G + C percentage (GC%) vs. GC skew.

The mitogenomes are biased toward Adenine (A) and Thymine (T), which is consistent with typical metazoan mitogenomes (Figure 2a; Supplementary Table S3). The overall A and T contents ranged from 70.72% to 87.27%. Among the PCGs, ATP8 showed the highest AT content (82.42–87.27%), whereas COI showed the lowest (70.89–72.57%). The value of AT-skew was consistently negative across all PCGs, indicating a higher content of T than A. In contrast, the value of GC-skew of each gene varied, being positive in the ND1, ND4, ND4L, and ND5 genes, while negative in most other genes.

The comparison was extended to all calliphorid subfamilies with more than one mitogenome available. We assessed the relationship between nucleotide content and compositional skew through linear regression analysis (Figure 2b,c). For both Rhiniinae and Calliphoridae, the regressions of AT% against AT-skew and GC% against GC-skew yielded very weak correlations (R^2^ < 0.02).

### 3.2. Codon Usage of PCGs

**Figure 3:**
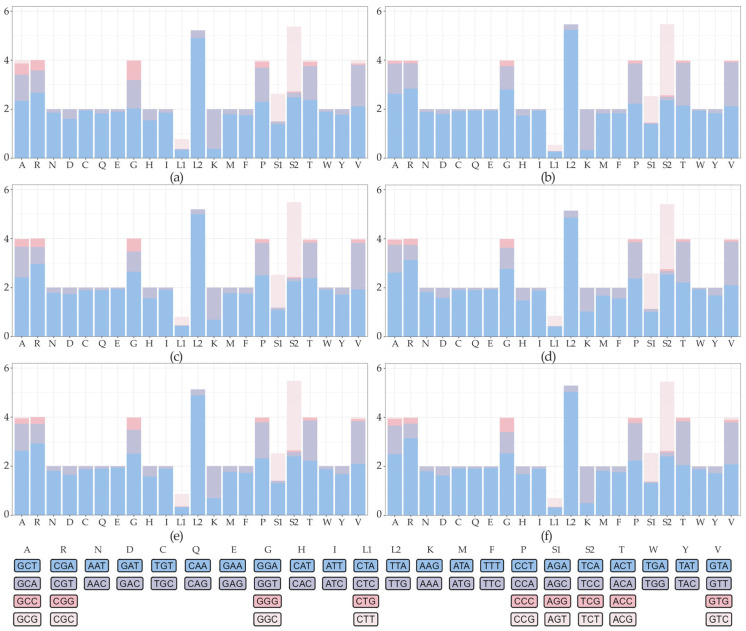
Relative synonymous codon usage (RSCU) in six Calliphoridae subfamilies. (**a**) Bengaliinae; (**b**) Calliphorinae; (**c**) Chrysomyinae; (**d**) Luciliinae; (**e**) Phumosiinae; and (**f**) Rhiniinae.

Analysis of relative synonymous codon usage (RSCU) across the six calliphorid subfamilies revealed a very high A + U bias (Figure 3). The most frequently used codons were exclusively A- or U-ending, with the leucine codon TTA (Leu2) being the most highly used (RSCU: 4.86–5.23). Other dominant codons included CGA (Arg, RSCU: 2.67–3.14), GGA (Gly, RSCU: 2.03–2.80), and GTA (Val, RSCU: 1.92–2.12).

Several G- or C-ending codons were completely absent (RSCU = 0) in multiple subfamilies. This included no arginine codon CGC in Luciliinae, Bengaliinae, Chrysomyinae, and Phumosiinae; no serine codon AGG in Bengaliinae and Phumosiinae; and no glycine codon GGC in Phumosiinae.

### 3.3. Analysis of Pi and Ka/Ks

To evaluate the patterns of genetic diversity within the major lineages of Calliphoridae, nucleotide diversity (Pi) for 13 protein-coding genes was calculated separately for each of the four subfamilies (Calliphorinae, Chrysomyinae, Luciliinae, and Rhiniinae; Supplementary Table S4). The diversity levels exhibited significant variation both across genes and among subfamilies, spanning from 0.029 to 0.110. The highest Pi value was detected in ND6 (0.110 in Chrysomyinae), followed by ATP8 (0.095 in Rhiniinae) and CYTB (0.093 in Rhiniinae). Despite their relatively short length, some regions showed a relatively high number of mutations (e.g., ATP8 in Rhiniinae [length: 165 bp; mutations: 62; Pi = 0.095]). In contrast, longer regions such as ND5 in Luciliinae (length: 1746 bp; mutations: 439; Pi = 0.073) presented a lower mutation density.

**Figure 4:**
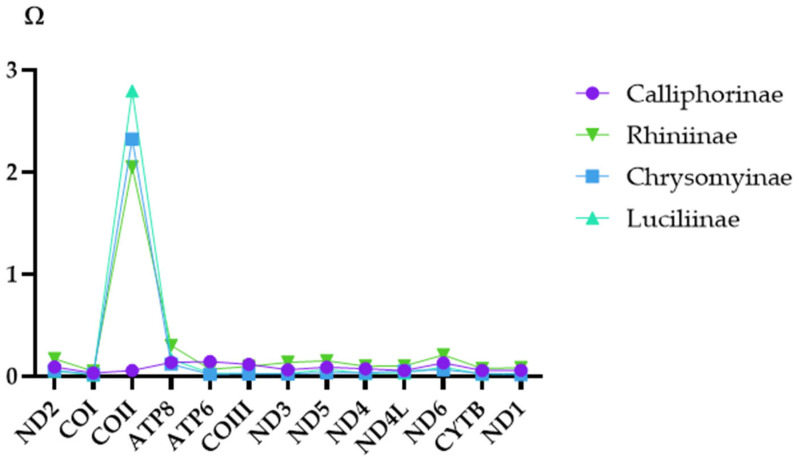
Distribution of *Ka/Ks* ratios for 13 mitochondrial protein-coding genes across four calliphorid subfamilies. COII showed ratios >1 in Chrysomyinae, Luciliinae, and Rhiniinae, contrasting with predominantly purifying selection in other genes.

The *Ka/Ks* ratios for 13 PCGs across the four subfamilies with more than one mitogenome were estimated: Calliphorinae, Chrysomyinae, Luciliinae, and Rhiniinae (Figure 4). Most genes exhibited *Ka*/*Ks* < 1, except for COII, which displayed *Ka/Ks* ratios substantially greater than 1 in the subfamilies Chrysomyinae, Luciliinae, and Rhiniinae, and reached as high as 2.80 in Luciliinae.

### 3.4. Phylogeny Reconstruction 

**Figure 5:**
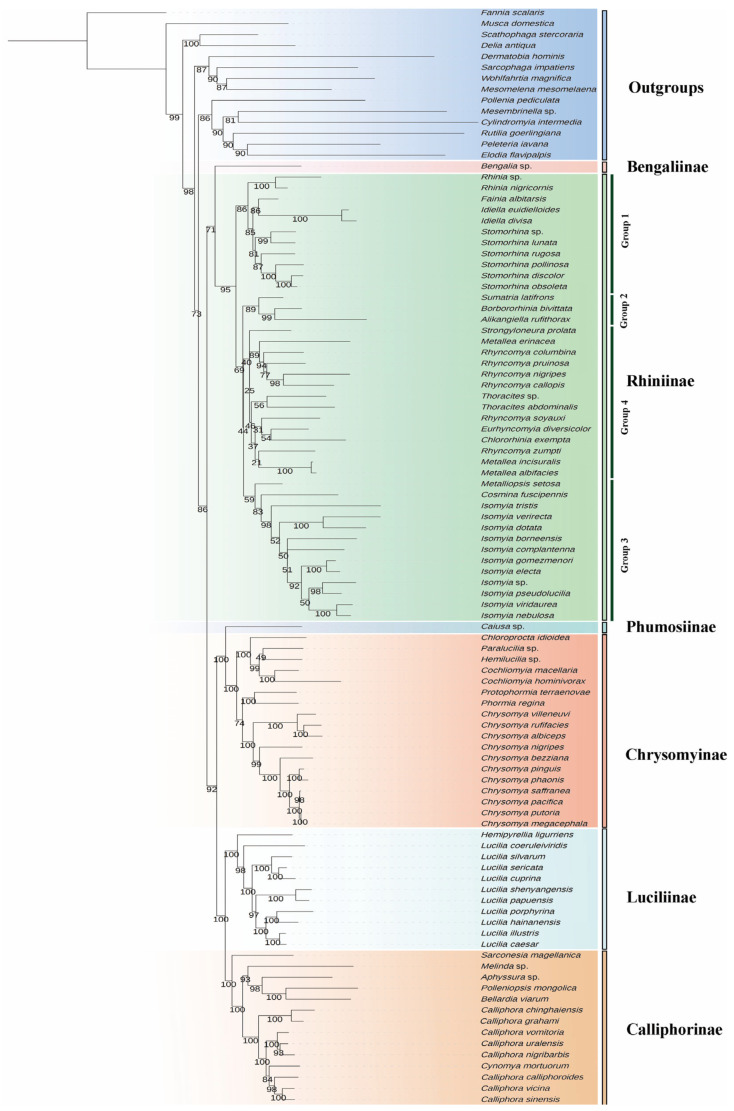
Maximum likelihood (ML) tree of the Calliphoridae inferred from mitochondrial genes.

**Figure 6:**
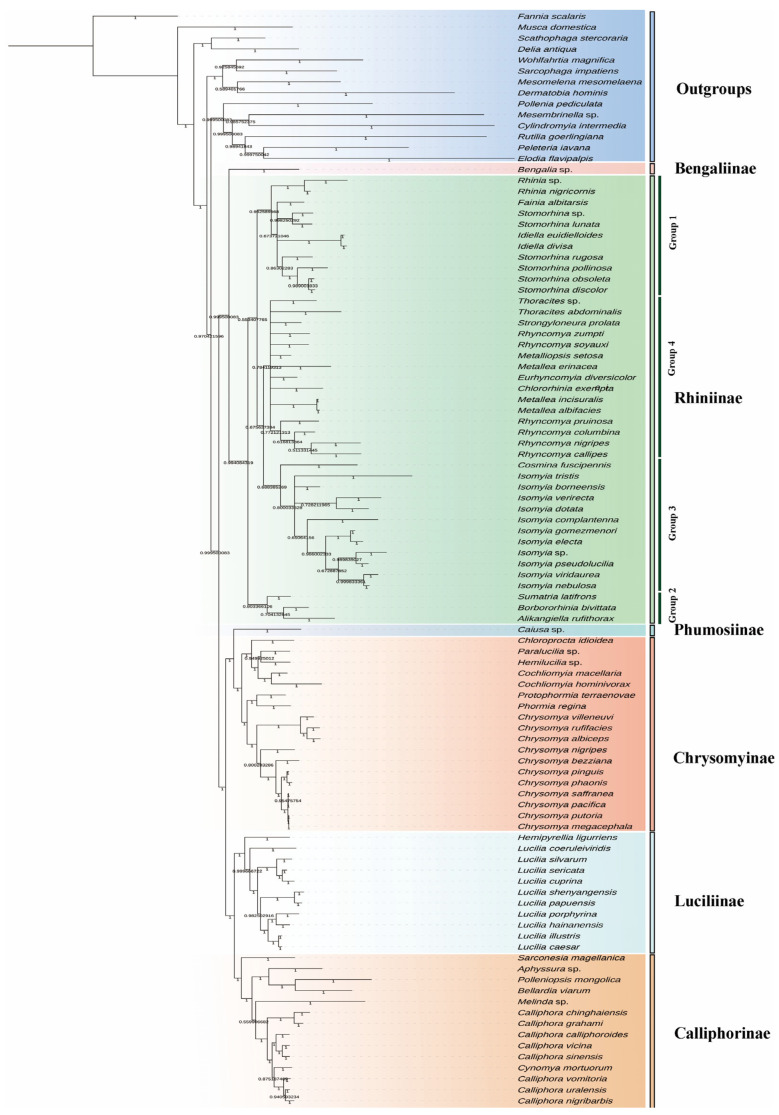
Bayesian inference (BI) tree of the Calliphoridae inferred from mitochondrial genes.

Phylogenetic analysis in the present study strongly supports the monophyly of Calliphoridae as a whole. Within Calliphoridae, all recognized subfamilies form distinct and highly supported monophyletic groups (Figure 5 and Figure 6). Calliphorinae and Luciliinae are fully supported as sister groups, which cluster together with (Chrysomyinae + Phumosiinae) with strong support (UFBS = 92, PP = 1.00). Bengaliinae and Rhiniinae are recovered as sister groups (UFBS = 71, PP = 1.00). Although this node receives only moderate support, the same sister-group relationship is evident, and together they form the sister lineage to the remaining calliphorids.

Within the Rhiniinae, four major groups are identified. However, the relationships among these groups differ slightly between analyses. In the ML tree, the topology is reconstructed as (Group 1 (Group 2 (Group 3, Group 4))), whereas the BI tree recovers (Group 2 (Group 1 (Group 3, Group 4))). In both analyses, Groups 3 and 4 form a stable sister relationship, while the placement of Groups 1 and 2 varies and receives relatively weak support. Group 1 comprises *Rhinia*, *Fainia*, *Idiella*, and *Stomorhina*, forming a distinct clade with modest support, and all three genera with more than one species sampled are strongly supported as monophyletic. Group 2 includes three genera, each sampled by one species, and the relationship is reconstructed as (*Sumatria*, (*Borbororhinia*, *Alikangiella*)). Within Group 3, *Isomyia* forms a highly supported monophyletic group (UFBS = 98, PP = 0.80), sistering to the genus *Cosmina*. A minor topological difference is observed in the placement of *Metalliopsis*, which is recovered as the basal lineage of Group 3 in the ML tree (UFBS = 59) but is placed within Group 4 in the BI analysis. The remaining genera fall into Group 4, although the relationships are not fully resolved, in terms of *Metallea* and *Rhyncomya* being paraphyletic.

## 4. Discussion

### 4.1. Mitogenome Characteristics

Mitogenomes of the six Rhiniinae species demonstrate typical features of insect mitogenomes. Among calliphorid subfamilies with more than one mitogenome available, nucleotide content and compositional skew show weak correlation, which suggests that strand asymmetry in calliphorid mitogenomes is largely independent of the overall nucleotide content. This aligns with previous findings that replication mechanisms and strand-specific mutational biases, rather than nucleotide composition alone, are the primary drivers of skew patterns in insect mitogenomes [42,43,44].

Consistent with the AT-rich nucleotide composition, a high A + U bias was observed in codon usage, with several G- or C-ending codons completely absent across multiple subfamilies. Despite these specific absences, the overall codon usage pattern was highly conserved across Calliphoridae, underscoring the presence of strong, lineage-wide compositional constraints.

While most mitochondrial protein-coding genes exhibited signals of purifying selection as found in many other calyptrate groups [1], the COII gene emerged as a distinct exception. Among the four calliphorid subfamilies examined, evidence of positive selection of COII was evident in three of them: Chrysomyinae (2.32), Luciliinae (2.80), and Rhiniinae (2.05). In contrast, in Calliphorinae (0.06), purifying selection was sustained. Given that COII encodes a subunit of cytochrome c oxidase, these findings suggest a history of adaptive evolution in this crucial oxidative phosphorylation component across multiple lineages [45,46,47].

### 4.2. Phylogenetic Implications for Rhiniinae

The phylogenetic analysis recovered four major groups within Rhiniinae. Group 1 corresponds well with the traditionally recognized tribe Rhiniini [5,7,48,49]. In addition, genera belonging to Groups 2–4 are grouped by morphological traits in tribe Cosminini [5,7,48,49]. Thus, the topology of the resulting tree is consistent with the traditional division of the group into two tribes, based on morphological characters. The classification and relationship of subfamilies within Calliphoridae have been controversial [1,50,51,52,53,54,55]. The present study, from the perspective of mitogenomics, lends support to the reclassification of Calliphoridae proposed by Yan et al. [1], and confirms the sister-group relationship between Rhiniinae and Bengaliinae, consistent with recent studies [2,56,57,58,59].

Previous studies of the Rhiniinae mostly focused on taxonomy, and the relationships within this subfamily have rarely been investigated [5]. Recently, Buenaventura et al. [60] sampled nine genera and reconstructed the phylogeny using ultraconserved element (UCE) data, while Beza-Beza et al. [58] sampled 14 genera and conducted phylogenetic reconstruction using anchored hybrid enrichment (AHE) data. The close relationship among genera belonging to tribe Rhiniini (*Rhinia*, *Fainia*, and *Stomorhina*) was identified by Buenaventura et al. [60], Beza-Beza et al. [58], and the present study, although the placement of *Fainia* differs in these studies. In Buenaventura et al. [60] and Beza-Beza et al.’s studies [58], *Fainia* is sister to *Rhinia* with high support. In the present study, the clade (*Fainia* + *Idiella*) is sister to *Stomorhina* with moderate support (UFBS = 85). *Eurhyncomyia* and *Rhyncomya* are closely related in all genomic studies. Interestingly, *Rhyncomya* is recovered as paraphyletic in the phylogeny reconstructed using mitogenomic and phylogenomic data, consistent with previous morphological studies, indicating that the genus has long been a residual assemblage subdivided into several species groups, some of which should be split into multiple genera [9,61,62]. The close relationship between *Isomyia*, *Cosmina*, *Eurhyncomyia*, and *Rhyncomya* is supported by UCE [60], AHE [58] and mitogenomic data, although it is not fully supported in UCE and the present mitogenomic trees. The present mitochondrial phylogeny offers an alternative perspective on the generic relationships within Rhiniinae, highlighting the value of the mitogenomic data in complementing nuclear-based phylogenetic inferences. Notably, the mitogenomic study consistently recovers a similar phylogeny of calliphorids as obtained by phylogenomic studies, demonstrating the reliability of the mitogenome for reconstructing phylogenetic relationships within this group.

## 5. Conclusions

This study documents the first complete or nearly complete mitogenomes of six Rhiniinae species. The mitogenomes show conserved gene content, strong AT bias, and similar codon usage patterns across calliphorid subfamilies. Most protein-coding genes are under purifying selection, whereas COII exhibits signatures of positive selection in multiple subfamilies. Phylogenetic analyses support the monophyly of Calliphoridae and recover four major lineages within Rhiniinae, consistent with morphological classification and recent phylogenomic studies. These results provide new mitogenomic resources and improve our understanding of evolutionary relationships within Rhiniinae.

## Figures and Tables

**Table 1:** Taxon sampling with GenBank accession numbers of data used in the present study.

Family	Subfamily	Species	Accession No.
Anthomyiidae	Anthomyiinae	*Delia antiqua*	NC_028226
Anthomyiidae	Scathophaginae	*Scathophaga stercoraria*	OR039275
Calliphoridae	Bengaliinae	*Bengalia* sp.	MK591038
Calliphoridae	Calliphorinae	*Aphyssura* sp.	OR771697
Calliphoridae	Calliphorinae	*Bellardia viarum*	NC_085207
Calliphoridae	Calliphorinae	*Calliphora calliphoroides*	NC_050875
Calliphoridae	Calliphorinae	*Calliphora chinghaiensis*	NC_029215
Calliphoridae	Calliphorinae	*Calliphora grahami*	NC_026996
Calliphoridae	Calliphorinae	*Calliphora nigribarbis*	NC_053679
Calliphoridae	Calliphorinae	*Calliphora sinensis*	NC_053662
Calliphoridae	Calliphorinae	*Calliphora uralensis*	NC_053677
Calliphoridae	Calliphorinae	*Calliphora vicina*	OY288238
Calliphoridae	Calliphorinae	*Calliphora vomitoria*	NC_028411
Calliphoridae	Calliphorinae	*Cynomya mortuorum*	MT628574
Calliphoridae	Calliphorinae	*Melinda* sp.	OP930889
Calliphoridae	Calliphorinae	*Polleniopsis mongolica*	NC_053676
Calliphoridae	Calliphorinae	*Sarconesia magellanica*	OR771696
Calliphoridae	Chrysomyinae	*Chloroprocta idioidea*	KT272777
Calliphoridae	Chrysomyinae	*Chrysomya albiceps*	NC_019631
Calliphoridae	Chrysomyinae	*Chrysomya bezziana*	NC_019632
Calliphoridae	Chrysomyinae	*Chrysomya megacephala*	NC_019633
Calliphoridae	Chrysomyinae	*Chrysomya nigripes*	NC_028412
Calliphoridae	Chrysomyinae	*Chrysomya pacifica*	KP_861632
Calliphoridae	Chrysomyinae	*Chrysomya phaonis*	NC_031381
Calliphoridae	Chrysomyinae	*Chrysomya pinguis*	NC_025338
Calliphoridae	Chrysomyinae	*Chrysomya putoria*	NC_002697
Calliphoridae	Chrysomyinae	*Chrysomya rufifacies*	NC_019634
Calliphoridae	Chrysomyinae	*Chrysomya saffranea*	NC_019635
Calliphoridae	Chrysomyinae	*Chrysomya villeneuvi*	MW592365
Calliphoridae	Chrysomyinae	*Cochliomyia hominivorax*	NC_002660
Calliphoridae	Chrysomyinae	*Cochliomyia macellaria*	KT272853
Calliphoridae	Chrysomyinae	*Hemilucilia* sp.	KT272860
Calliphoridae	Chrysomyinae	*Paralucilia* sp.	KT272861
Calliphoridae	Chrysomyinae	*Phormia regina*	NC_026668
Calliphoridae	Chrysomyinae	*Protophormia terraenovae*	OX596080
Calliphoridae	Luciliinae	*Hemipyrellia ligurriens*	NC_019638
Calliphoridae	Luciliinae	*Lucilia caesar*	NC_028057
Calliphoridae	Luciliinae	*Lucilia coeruleiviridis*	NC_029486
Calliphoridae	Luciliinae	*Lucilia cuprina*	PP297113
Calliphoridae	Luciliinae	*Lucilia hainanensis*	MW592363
Calliphoridae	Luciliinae	*Lucilia illustris*	NC_028056
Calliphoridae	Luciliinae	*Lucilia papuensis*	NC_053672
Calliphoridae	Luciliinae	*Lucilia porphyrina*	NC_019637
Calliphoridae	Luciliinae	*Lucilia sericata*	MW255540
Calliphoridae	Luciliinae	*Lucilia shenyangensis*	NC_059913
Calliphoridae	Luciliinae	*Lucilia silvarum*	MT872670
Calliphoridae	Phumosiinae	*Caiusa* sp.	OP930888
Calliphoridae	Rhiniinae	*Alikangiella rufithorax*	MG967831
Calliphoridae	Rhiniinae	*Borbororhinia bivittata*	JQ246691
Calliphoridae	Rhiniinae	*Chlororhinia exempta*	MG967840
Calliphoridae	Rhiniinae	*Cosmina fuscipennis*	KR820754
Calliphoridae	Rhiniinae	*Eurhyncomyia diversicolor*	MN411268
Calliphoridae	Rhiniinae	*Fainia albitarsis*	MN411166
Calliphoridae	Rhiniinae	*Idiella divisa*	KY031806
Calliphoridae	Rhiniinae	*Idiella euidielloides*	MT888829
Calliphoridae	Rhiniinae	*Isomyia borneensis*	KT276327
Calliphoridae	Rhiniinae	*Isomyia complantenna*	KY031773
Calliphoridae	Rhiniinae	*Isomyia dotata*	MT888830
Calliphoridae	Rhiniinae	*Isomyia electa*	KY031768
Calliphoridae	Rhiniinae	*Isomyia gomezmenori*	JF439553
Calliphoridae	Rhiniinae	*Isomyia nebulosa*	NC083970
Calliphoridae	Rhiniinae	*Isomyia pseudolucilia*	KY031770
Calliphoridae	Rhiniinae	*Isomyia* sp.	MF804688
Calliphoridae	Rhiniinae	*Isomyia tristis* *	PX508347
Calliphoridae	Rhiniinae	*Isomyia verirecta*	KY031774
Calliphoridae	Rhiniinae	*Isomyia viridaurea*	MT888831
Calliphoridae	Rhiniinae	*Metallea albifacies* *	PX549227
Calliphoridae	Rhiniinae	*Metallea erinacea*	GQ409337, GQ409405, GQ409135, GQ409074
Calliphoridae	Rhiniinae	*Metallea incisuralis*	HM399351
Calliphoridae	Rhiniinae	*Metalliopsis setosa*	OR515114
Calliphoridae	Rhiniinae	*Rhinia nigricornis* *	PX549224
Calliphoridae	Rhiniinae	*Rhinia* sp.	JQ246743
Calliphoridae	Rhiniinae	*Rhyncomya callopis* *	PX549228
Calliphoridae	Rhiniinae	*Rhyncomya columbina*	MN868846
Calliphoridae	Rhiniinae	*Rhyncomya nigripes*	GQ409423, GQ409092
Calliphoridae	Rhiniinae	*Rhyncomya pruinosa* *	PX549225
Calliphoridae	Rhiniinae	*Rhyncomya soyauxi*	JQ246744, JQ246693
Calliphoridae	Rhiniinae	*Rhyncomya zumpti*	JQ246693
Calliphoridae	Rhiniinae	*Stomorhina discolor*	GQ409375, GQ409441, GQ409110
Calliphoridae	Rhiniinae	*Stomorhina lunata*	OW121746
Calliphoridae	Rhiniinae	*Stomorhina obsoleta*	NC085206
Calliphoridae	Rhiniinae	*Stomorhina pollinosa*	HM421627
Calliphoridae	Rhiniinae	*Stomorhina rugosa* *	PX549226
Calliphoridae	Rhiniinae	*Stomorhina* sp.	OP930885
Calliphoridae	Rhiniinae	*Strongyloneura prolata*	MT888841
Calliphoridae	Rhiniinae	*Sumatria latifrons*	MG967924
Calliphoridae	Rhiniinae	*Thoracites abdominalis*	MG968089
Calliphoridae	Rhiniinae	*Thoracites* sp.	JQ246745, JQ246694
Fanniidae		*Fannia scalaris*	NC_053661
Mesembrinellidae		*Mesembrinella* sp.	KT272778
Muscidae		*Musca domestica*	NC_024855
Oestridae		*Dermatobia hominis*	NC_006378
Polleniidae		*Pollenia pediculata*	OZ199120
Sarcophagidae		*Mesomelena mesomelaena*	KY003227
Sarcophagidae		*Sarcophaga impatiens*	NC_017605
Sarcophagidae		*Wohlfahrtia magnifica*	KU578263
Tachinidae		*Cylindromyia intermedia*	NC_060867
Tachinidae		*Elodia flavipalpis*	NC_018118
Tachinidae		*Peleteria iavana*	NC_063086
Tachinidae		*Rutilia goerlingiana*	NC_019640

* Newly sequenced mitogenomes in this study.

## Data Availability

Data Availability Statement: The six newly sequenced mitogenomes were submitted to the GenBank database under the accession numbers of *Isomyia tristis* (PX508347), *Metallea albifacies* (PX549227), *Rhinia nigricornis* (PX549224), *Rhyncomya callopis* (PX549228), *Rhyncomya pruinosa* (PX549225), and *Stomorhina rugosa* (PX549226).

## Supplementary Materials

The following supporting information can be downloaded at https://www.mdpi.com/article/10.3390/ani16091289/s1. Table S1: Collection information for the six newly sequenced Rhiniinae species; Table S2: Collection information of specimens used for DNA extraction in the present study. A “*” represents the specimens sequenced in this study; Table S3: Nucleotide composition (A + T content) of the 13 mitochondrial protein-coding genes across six subfamilies of Calliphoridae; Table S4: Nucleotide diversity (Pi) and number of mutations of 13 mitochondrial protein-coding genes across four subfamilies of Calliphoridae. (a) Nucleotide diversity (Pi) calculated for each gene within each subfamily. (b) Number of variable (mutated) sites detected in each gene and subfamily.
